# Calcium Channels: Noteworthy Regulators and Therapeutic Targets in Dermatological Diseases

**DOI:** 10.3389/fphar.2021.702264

**Published:** 2021-08-19

**Authors:** Min Wang, Yaoxiang Sun, Linli Li, Peipei Wu, Ocansey DKW, Hui Shi

**Affiliations:** ^1^ Jiangsu Key Laboratory of Medical Science and Laboratory Medicine, Institute of Stem Cell, School of Medicine, Jiangsu University, Zhenjiang, China; ^2^ Department of Clinical Laboratory, The Affiliated Yixing Hospital of Jiangsu University, Yixing, China; ^3^ Directorate of University Health Services, University of Cape Coast, Cape Coast, Ghana

**Keywords:** calcium channels, dermatologic disease, transient receptor potential, therapeutic target, second messenger

## Abstract

Dysfunctional skin barrier and impaired skin homeostasis may lead to or aggravate a series of dermatologic diseases. A large variety of biological events and bioactive molecules are involved in the process of skin wound healing and functional recovery. Calcium ions (Ca^2+^) released from intracellular stores as well as influx through plasma membrane are essential to skin function. Growing evidence suggests that calcium influx is mainly regulated by calcium-sensing receptors and channels, including voltage-gated, transient potential receptor, store-operated, and receptor-operated calcium channels, which not only maintain cellular Ca^2+^ homeostasis, but also participate in cell proliferation and skin cell homeostasis through Ca^2+^-sensitive proteins such as calmodulin (CaM). Furthermore, distinct types of Ca^2+^ channels not merely work separately, they may work concertedly to regulate cell function. In this review, we discussed different calcium-sensing receptors and channels, including voltage-gated, transient receptor potential, store-operated, and receptor-operated calcium channels, particularly focusing on their regulatory functions and inherent interactions as well as calcium channels-related reagents and drugs, which is expected to bridge basic research and clinical applications in dermatologic diseases.

## Introduction

The skin is the largest and heaviest organ of the body and is subjected to a wide spectrum of traumatic injury. During the process of wound healing, a large variety of bioactive molecules are involved. Calcium ions (Ca^2+^) are one of the most diverse signaling mediators and intracellular second messengers, and contribute to establish and maintain the skin architecture and homeostasis (Pillai et al., 1990). Calcium is normally sequestered outside the cell or stored in organelles such as the endoplasmic reticulum (ER) and sarcoplasmic reticulum (SR); in contrast, calcium levels are low in the cytoplasm, leading to the generation of a calcium concentration gradient. The calcium gradient with peaking calcium concentrations in the stratum granulosum and a steep drop-off in the stratum corneum in epidermis is important to maintain skin barrier function (Elsholz et al., 2014). When the skin is impaired, Ca^2+^-sensing receptor (CaSR) conveys the signaling of extracellular Ca^2+^ to interior of the cell through different calcium channels, causing skin cells adhesion, differentiation, and survival.

There are generally two classes of calcium channels, calcium entry channels, which allow extracellular Ca^2+^ to enter intracelluar cells, and calcium release channels, which transfer Ca^2+^ from intracellular stores to the cytoplasm. Calcium entry channels include voltage-gated calcium channels (VGCCs), ligand-gated calcium channels, store operated calcium channels (SOCCs) and transient receptor potential (TRP) channels Calcium release channels include ryanodine receptors (RyRs) and inositol 1,4,5-trisphosphate receptors (IP_3_Rs) (Greenberg, 1997). It is gradually realized that calcium channels are involved in skin function maintenance under physiological and pathological conditions. The purpose of this review is to summarize the current state of calcium-sensing receptors and channels research, their roles in diverse pathological conditions, and the current contribution of calcium pathway-related drugs, which could help to develop new research and therapeutic strategy in intractable skin diseases.

## Calcium-Sensing Receptors and Channels Mediated Ca^2+^ Signaling in Skin Function Maintenance

**FIGURE 1:**
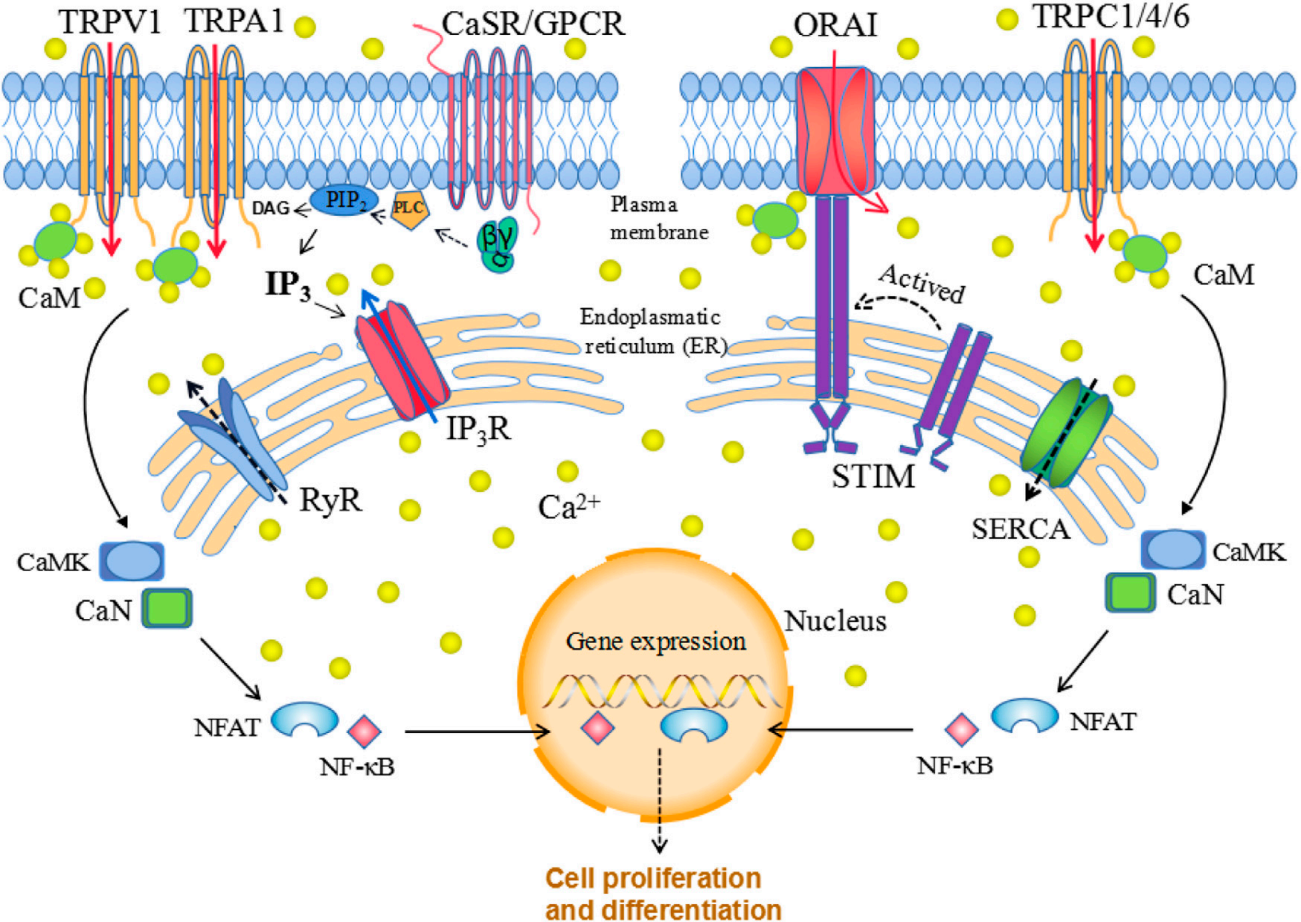
Cellular calcium homeostasis. The calcium-sensing receptor (CaSR), which is a G protein-coupled receptor (GPCR), senses changes in extracellular calcium concentrations, leading to phospholipase C (PLC) activation; activated PLC then hydrolyzes phosphatidylinositol-4,5-bisphosphate (PIP_2_), generating inositol 1,4,5-trisphosphate (IP_3_) and diacylglycerol (DAG). The binding of IP_3_ to the IP_3_ receptor (IP_3_R) located in the ER allows Ca^2+^ release from the ER. When the ER Ca^2+^ stores are depleted, this is sensed by STIM family proteins localized in the ER membrane, which translocate to the plasma membrane and interact with ORAI, thereby triggering selective store-operated Ca^2+^ entry (SOCE). Similarly, release of Ca^2+^ through ryanodine receptors (RyRs) is pumped back into the endoplasmic reticulum calcium through ATPase (SERCA). Elevated [Ca^2+^]i regulate gene expression via Ca^2+^/CaM pathway. The main downstream targets: Ca^2+^/CaM-kinases (CaMK) and calcineurin (CaN), could regulate transcription factors such as NFAT and NFκB, to influence cell function (Figure 1).

Ca^2+^ signaling is strictly regulated while both CaSR and calcium channels are essential components that allow Ca^2+^ to enter the cell in response to various stimuli. Signal transduction via CaSRs occurs mainly through the Gɑq pathway. G-protein activation further stimulates phospholipase C (PLC), which hydrolyzes phosphatidylinositol-4,5-bisphosphate (PIP_2_), generating inositol 1,4,5 triphosphate (IP_3_) and diacylglycerol (DAG). IP_3_ diffuses through the cytoplasm and binds to IP_3_ receptors (IP_3_Rs) on the surface of the ER/SR, thereby promoting Ca^2+^ transportation from these compartments to the cytoplasm. In addition, released Ca^2+^ and DAG stimulate protein kinase C (PKC), which phosphorylates other molecules and further generate productions (Dhyani et al., 2020). Elevated [Ca^2+^]_i_ regulate cell cycle via Ca^2+^/CaM pathway. The main downstream targets-Ca^2+^/CaM-kinases (CaMK) and calcineurin (CaN), could regulate transcription factors such as NFAT and NFκB, to influence cell function (Figure 1). During the process, various calcium channels may act independently or in concert to assist the Ca^2+^ signaling transduction and mediate diverse biological functions.

### Voltage-Gated Calcium (Ca_v_) Channels

Electrical activities in the body are mostly transmitted via the plasma membrane. Voltage-gated calcium (Ca_V_) channels are transmembrane protein activated by depolarization of membrane potential (Andrade et al., 2019) and responsible for converting membrane electrical signals to intracellular Ca^2+^ transients (Wu et al., 2017), leading to the activation of multiple physiological events. Ca_V_s consist of a complex of α1 subunit associated with β, γ, and α2δ subunits (Zamponi et al., 2015). The α1 subunits contain four homologous domains with six transmembrane segments (Mikami et al., 1989), where transmembrane segments S1–S4 form the voltage-sensing module, and S5 and S6 form the pore1 (Tang et al., 2016). When stimulated, cell membranes depolarize and action potentials occur. Voltage-sensing module senses the changes of potential and mediate Ca^2+^ influx (Catterall et al., 2011). Most physiological phenomena, including muscle contraction, synaptic transmission, hormone secretion, gene expression, and cell death, are stimulated by Ca^2+^, which functions as a universal second messenger. At the same time, VGCCs was demonstrated to provide Ca^2+^ with access to the intracellular environment (Catterall, 2011; Wu et al., 2017).

There are 10 subtypes of VGCCs, which can be classified into three subfamilies: Ca_v_1, Ca_v_2, and Ca_v_3. The Ca_v_1 channels, designated as L-type calcium channels, are activated at high voltage to conduct large and long-lasting ion currents (Tsien et al., 1988). Ca_v_2 channels can be classified as P/Q-type, N-type, and R-type based on their current properties and inhibition (Nowycky et al., 1985; Randall and Tsien, 1995). Meanwhile, Ca_v_3 channels, designated as T-type calcium channels, are activated at low voltage and conduct transient currents (Carbone and Lux, 1984; Nowycky et al., 1985). Das et al. (2012) identified that normal melanocytes and melanoma cells both express Ca_v_1, Ca_v_2 channels, while only melanoma cells express Ca_v_3 (T-type) channels. Das et al. (2012) also found that T-type channel blocker kurtoxin could reduce viability and proliferation of the melanoma cells, encouraging T-type channels as potential pharmacological therapy targets. VGCCs are also found to exist on epidermal keratinocytes. Denda et al. (2006) demonstrated that the influx of Ca^2+^ ions into epidermal keratinocytes delay the barrier recovery, while the administration of nifedipine, verapamil, or R-(+)-BAY K8644, inhibitors of VGCC, accelerate the barrier recovery. When skin barrier is impaired, its electric potential is changed, generating negative electric potentials to promote skin barrier repair (Denda and Kumazawa, 2002). These findings indicate that VGCCs are associated with skin homeostasis and play a negative role in skin barrier recovery.

### Transient Receptor Potential Channels

Transient receptor potential (TRP) genes were firstly described in Drosophila melanogaster. The study identified that a visually impaired mutant fly showed a transient instead of sustained response to steady light (Cosens and Manning, 1969). The trp gene as well as the structure and localization of the trp protein was identified 2 decades later by Montell and Rubin (Montell and Rubin, 1989). In humans the first TRP-encoding gene was only reported in 1995 (Wes et al., 1995). Since then, approximately 30 TRP-related genes and 27 different TRP channels in human have been identified (Li, 2017). TRP channels act as communication stations for cells and their functions are cell type-dependent. In neurons, for instance, activated TRP channels may cause depolarization and stimulate electric potential generation. However, in non-excitable cells, TRP channels regulate intracellular calcium concentrations, which are related to keratinocytes proliferation and differentiation to influence skin barrier (Moran et al., 2018). TRP channels were originally described as “polymodal cellular sensors” (Clapham, 2003; Damann et al., 2008; Vay et al., 2012); but they are now considered to be “promiscuous pleiotropic molecules” that can also be triggered by multiple physical, chemical, and other relevant factors (Ramsey et al., 2006; Vriens et al., 2008; Vriens et al., 2009).

**FIGURE 2:**
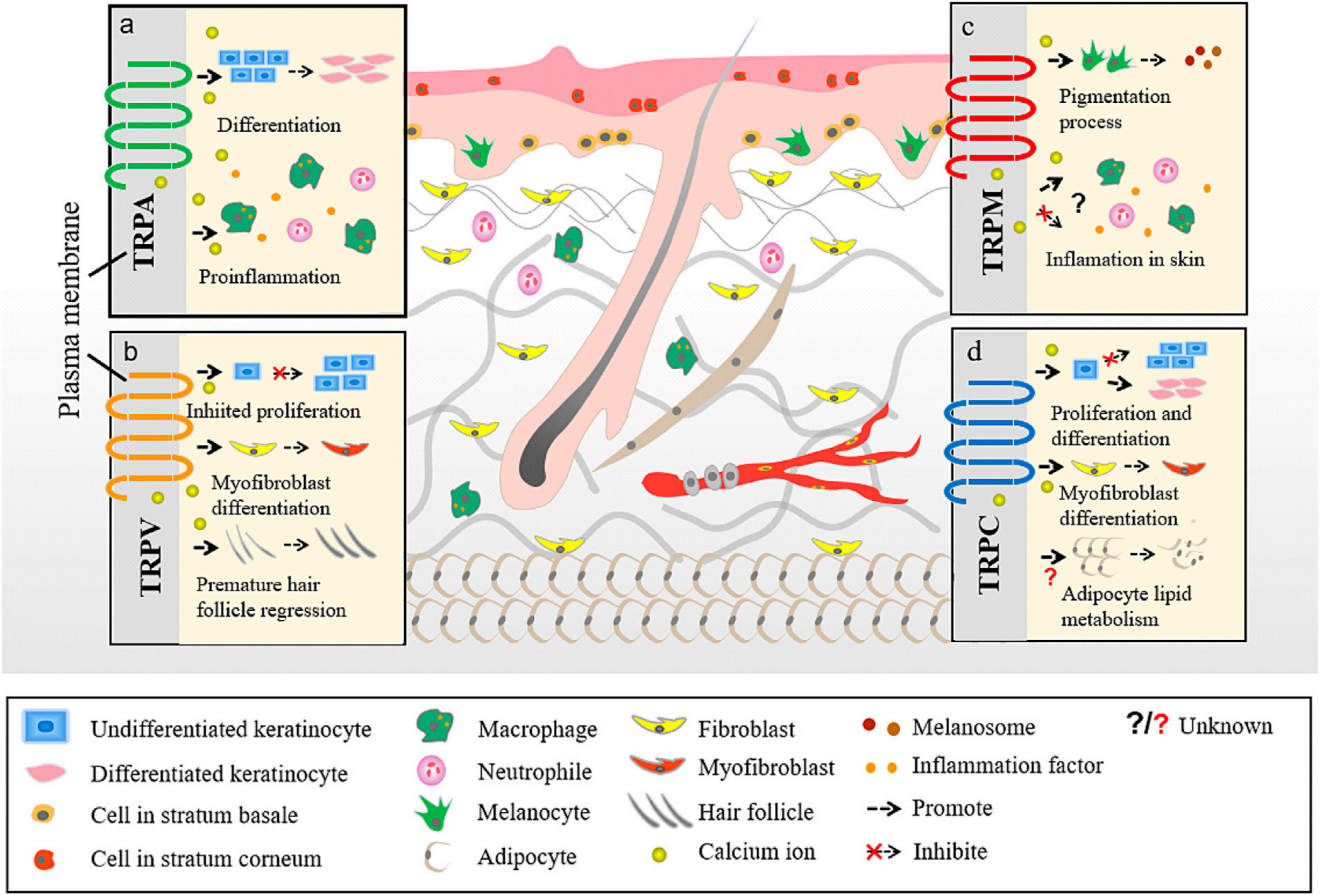
Different TRP channels participate in different skin homoeostasis and barrier function. a. The interaction between TRPA and skin. b. The interaction between TRPV and skin. c. The interaction between TRPM and skin. d. The interaction between TRPC and skin.

TRP channels can be classified into several types depending on their structure. There are seven TRP subfamilies: TRPC (canonical), TRPV (vanilloid), TRPM (melastatin), TRPA (ankyrin), TRPP (polycystin), TRPML (mucolipin), and TRPN (Drosophila NompC) (Goel et al., 2002; Montell et al., 2002; Atoyan et al., 2009). An eighth TRP family was recently identified in yeast and named as TRPY (Li et al., 2017). Based on their homology to Drosophila TRP, TRP subfamilies are classified into group 1 TRP channels, which shows the greatest similarity with Drosophila TRP channels and includes TRPC, TRPV, TRPM, and TRPA; and group 2 TRP channels, - TRPP and TRPM - that are more distantly related to the TRP channels of Drosophila (Li et al., 2017). Emerging evidence suggests that several TRP channels (Alexander et al., 2013) are involved in cutaneous disorders, such as atopic dermatitis (AD), psoriasis, acne vulgaris, various forms of dermatitis, hair growth disorders, and cutaneous malignancies (Toth et al., 2014) (Figure 2).

#### TRPC

The TRPC family consists of seven members (TRPC1-7), which are highly related to Drosophila TRP channels. TRPCs are non-selective cation channels that are expressed in both excitable and non-excitable cells and regulate intracellular Ca^2+^ influx in response to numerous physiological or pathological stimuli (Feng, 2017). Increased Ca^2+^ influx causes membrane depolarization and cytosolic Ca^2+^ concentration ([Ca^2+^]c) elevation, both causing critical effect on cell function (Wang et al., 2020). Studies have reported the role of TRPCs in diabetic kidney disease and myocardial injury (He et al., 2017; Ilatovskaya et al., 2018).Blocking of TRPC activity protects the function of podocytes and cardiac cells, indicating TRPC as a promising target for pharmacologic intervention. Moreover, TRPCs are also expressed in human epithelial cells in the epidermis (Tu et al., 2005) and are shown to play a role in Darier’s disease by regulating keratinocyte proliferation and differentiation (Beck et al., 2008).

During fetal development, TRPC1 expression is higher in the brain than in the liver and kidney, while in the adult it is primarily active in the heart, testes, ovary, and numerous regions of the brain (Strubing et al., 2001). In humans, TRPC2 is classified as a pseudogene (Montell et al., 2002). In the epidermis, TRPC1 contributes to keratinocytes differentiation by regulating Ca^2+^ influx (Cai et al., 2006). TRPC1 on the surface of endothelial cells was demonstrated to interact with both soluble α-Klotho, which is a pleiotropic molecule with multiple effects (e.g., antioxidant and vascular-protective), and vascular endothelial growth factor (VEGF)/VEGF receptor 2, which regulates Ca^2+^ influx and plasma membrane permeability, thereby contributing to the maintenance of endothelial cell integrity (Mazzotta et al., 2017). Dysregulated TRPC1/3 mRNA in adipocytes cultured from human subcutaneous white adipose tissue has been linked to obesity, diabetes, and cardiovascular disease (Ondrusova et al., 2017). In addition, the expression of TRPC3 has been associated with other skin diseases, such as melanoma, which exhibits an extremely poor prognosis owing to its rapidly progressive and highly metastatic nature. The pharmacological inhibition of TRPC3 by Pyr3, a pyrazol compound, was shown to decrease melanoma cell proliferation and migration, and knockdown of TRPC3 reduced melanoma cell proliferation (Oda et al., 2017).

The *trans*-differentiation of fibroblasts into myofibroblasts induced by damaged conditions can secrete extracellular matrix to promote wound healing. TRPC6 expression, mediated by noncanonical TGF-β signaling through p38/SRF, activates the Ca^2+^-responsive protein phosphatase calcineurin, leading to the induction of myofibroblast *trans*-differentiation (Davis et al., 2012). In the epidermis, TRPC6 is primarily expressed in keratinocytes of the stratum spinosum and stratum granulosum, where keratinocytes undergo differentiation, but not in the basal layer, which suggests that TRPC6 may regulate Ca^2+^-induced keratinocyte differentiation (Muller et al., 2008). Furthermore, hyperforin, a major component of St. John’s wort, could also activate TRPC6 to enhance Ca^2+^ entry and ATP-Ca^2+^ signaling in keratinocytes, thereby contributing to cutaneous wound healing (Takada et al., 2017). Similarly, there is also *in vivo* and *in vitro* evidence that the TRPC4 protein is expressed in the cell membrane and cytoplasm of gingival keratinocytes and regulate CaSR-induced increase in intracellular [Ca^2+^] (Fatherazi et al., 2007).

Additionally, some members of the TRPC family can also interact with each other and may even function as a complex; for instance, TRPC3 can act as a STIM1-dependent SOCC only, by assembling with TRPC1, while TRPC6 functions as a STIM1-dependent channel only, in the presence of TRPC4 (Yuan et al., 2007). STIM1 regulates TRPC1 and TRPC4, which also mediate store-operated Ca^2+^ entry, and influence keratinocytes differentiation and formation of epidermis barrier via changing Ca^2+^ concentration (Lee and Lee, 2018).

#### TRPV

TRPVs are sensitive to various tissue-damaging signals and their activation is generally perceived as pain. It is also demonstrated that thermo-TRP channels such as TRPV1, TRPV2, TRPV3, and TRPV4 can be activated by heat. The TRPV subfamily consists of six members (TRPV1-6) (Li et al., 2017). The immunoreactivity of TRPV1-4 has been differentially identified on basal and supra-basal cells of healthy human skin, and these channels have been proposed to function as thermo-sensory receptors (Chung et al., 2004; Radtke et al., 2011). TRPV5 and TRPV6 proves to be epithelial calcium ion channels, while TRPV1-4 which are referred to as nociceptor that sense the damaging signals (Satheesh et al., 2016).

TRPV1 is a sensor of noxious heat, capsaicin, and protons (low pH) (Caterina et al., 1997), and could also respond to UV stimulation. Hair shaft elongation and matrix keratinocyte proliferation can be inhibited by TRPV1 stimulation and lead to premature hair follicle regression and cell apoptosis (Toth et al., 2009). The application of AEA, an endocannabinoid anandamide, can suppress epidermal cell proliferation and induce cell death due to Ca^2+^ entry through TRPV1 and the concomitant elevation of the intracellular Ca^2+^ concentration (Toth et al., 2011). A recent study reported that the inhibition of TRPV1 in differentiated human primary keratinocytes abrogated proteinase-activated receptor-2 (PAR-2) activating peptide SLIGKV, which evoked Ca^2+^ store depletion and the production of inflammatory mediators (Gouin et al., 2018). These findings indicate that hyperactive TRPV1 may inhibit keratinocytes proliferation and promote inflammation.

TRPV2 is regulated by temperature, ligands such as probenecid and cannabinoids, and lipids. TRPV2 shares a high sequence identity with TRPV1 (>50%), but exhibits a higher temperature threshold and sensitivity for activation than TRPV1 (Zubcevic et al., 2016). Unlike TRPV1, there is little evidence for the presence of TRPV2 in human epithelial cells, except for one study that demonstrated the presence of TRPV2 in human skin by immunostaining. Moreover, there is no existing data regarding the physiological role of TRPV2 in epithelial cells (Radtke et al., 2011). However, in a vitro wound healing model of rats, in a culture model of wound healing, compounds targeting TRPV2 channels were suggested to ameliorate excessive wound contraction through the inhibition of TGF-β1 release and the differentiation of dermal fibroblasts (Ishii et al., 2018).

TRPV3 is responsive to warm temperatures (<33°C) and is predominantly expressed in skin keratinocytes, where it mediates warm and pain sensation (Peier et al., 2002; Smith et al., 2002). A study showed that activated TRPV3 increases thymic stromal lymphopoietin (TSLP), nerve growth factor, prostaglandin E2, and interleukin (IL)-33 production in human keratinocytes and induces scratching behavior in mice (Seo et al., 2020). The stimulation of TRPV3 can also trigger a strong proinflammatory response via the NF-κ B pathway (Szollosi et al., 2018). Studies have also reported that the expression of TRPV3 and TSLP is increased in the tissues of pruritic burn scars (Park et al., 2017), and that TRPV3 induces myofibroblast differentiation, collagen production, and TSLP expression through the TRPV3-Smad2/3 signaling pathway (Um et al., 2020). These results suggest a direct interaction between TRPV3 and pruritus diseases, hence the inhibition of TRPV3 may be a promising therapy target.

Similar to TRPV3, TRPV4 can also be activated by warm temperature (Guler et al., 2002; Watanabe et al., 2002) and was initially reported to be an osmo- or mechano-sensor (Liedtke et al., 2000). TRPV4 is widely expressed throughout the body (Sokabe et al., 2010). In TRPV4-deficient mice, the epidermal barrier is impaired, and displays characteristics such as leaky cell-cell junctions, non-physiological actin rearrangements, and insufficient stratification (Kida et al., 2012). Ammar et al. (Boudaka et al., 2020) found that both cell migration and proliferation were slower in wild-type esophageal keratinocytes than in those with TRPV4 knockout cells. In addition, Adapala et al. (2013) found TRPV4 as a requirement for the TGF-β1-induced differentiation of cardiac fibroblasts into myofibroblasts. The application of AB159908, a TRPV4-specific antagonist, or siRNA knockdown of TRPV4 significantly inhibited TGFβ1-induced differentiation. The role of TRPV4 in cardiac fibroblasts has been demonstrated, but whether TRPV4 functions in dermal fibroblasts of skin, or participates in wound healing and skin barrier homeostasis remain questions that require further studies.

TRPV5 and TRPV6, both calcium-selective channels, are expressed at the apical membrane of Ca^2+^-transporting epithelia, and serve as entry channels in transepithelial Ca^2+^ transport (Flores-Aldama et al., 2020). TRPV6, which is also involved in skin barrier formation and function, was shown to play a crucial role in the terminal differentiation process induced by elevated extracellular Ca^2+^ concentrations (Lehen'Kyi et al., 2007). Furthermore, keratinocytes lacking TRPV6 exhibited a loss of close contacts between adjacent cells and the ability to flatten (Elsholz et al., 2014).

#### TRPA

TRPA1, an exclusive member of the TRPA family, is widely expressed in sensory neurons and in non-neuronal cells such as epithelial cells and hair cells, and can be activated by noxious external stimuli and low temperature (Talavera et al., 2020). As the members of the TRPV subfamily, TRPA1 is also involved in regulating epidermal cell biological function (Toth et al., 2014). For instance, it has been demonstrated that the topical application of TRPA1 agonists could accelerate barrier recovery from skin permeability (Denda et al., 2010). A recent study also showed that inhibitors of TRPA1 and a neurogenic inflammatory peptide released following TRPA1 activation- CGRP, reduced skin edema and the levels of proinflammatory cytokines (Achanta et al., 2018). In addition, activated TRPA1 has been associated with some of the symptoms of irritant contact dermatitis, such as pain, neurogenic inflammation, and, possibly, itching (Noroes et al., 2019). All these findings suggest that TRPA1 contributes to skin injury and inflammation.

#### TRPM

As intrinsic membrane proteins, the TRPM subfamily is divided into eight variable types, i.e., TRPM1-8 (Clapham et al., 2001). TRPMs are Ca^2+^-permeable cation channels except for TRPM4 and 5 (Ullrich et al., 2005; Hof et al., 2016). Human epidermal melanocytes express TRPM1, which is shown to be essential for the pigmentation process (Devi et al., 2009; Oancea et al., 2009). Just as TRPV1 is a key heat detector, TRPM8 is mainly sensitive to environmental cold (Moran, 2018). In contrast to TRPA1, TRPM8 does not seem to contribute to phthalate-induced skin hypersensitivity, as it is not activated by dibutyl phthalate (Kurohane et al., 2013). To date, TRPM8 has not been linked to skin homeostasis or dermatitis (Caterina, 2014). However, its activation can suppress chemically evoked irritation and inhibit TRPV1-mediated CGRP release in colon tissue (Ramachandran et al., 2013), and can also induce an increase in the level of some proinflammatory cytokines in the blood of normotensive rats (Kozyreva et al., 2016).

### Store-Operated Ca^2+^ Channels

As the major store of intracellular Ca^2+^, ER Ca^2+^ release could be triggered by IP_3_,a critical second messenger, causing ER Ca^2+^ release into the cytosol. (Putney, 2015). proposed a ‘capacitative Ca^2+^ entry’ hypothesis that the emptying of Ca^2+^ stores itself activates Ca^2+^ channels in the plasma membrane to help refill the stores. The hypothesis later renamed store-operated Ca^2+^ entry, or SOCE (Lewis, 2007). Study also identified that SOCE not only provides Ca^2+^ for refilling stores, but can itself generate sustained Ca^2+^ signals that control such essential functions as gene expression, cell metabolism and exocytosis (Parekh and Putney., 2005). Stromal interaction molecule (STIM) proteins (STIM1 and STIM2), are identified as Ca^2+^ sensors for SOCE. Once Ca^2+^ store depletion is sensed, STIM will translocate to plasma membrane and activate ORAI Ca^2+^ channels, the store-operated Ca^2+^ channel (Umemura et al., 2014; Prakriya and Lewis, 2015). Moreover, Hooper and Soboloff, 2015 found SOCE can be promoted by STIM-activating enhancers, such as STIMATE, which is located in the ER membrane, can translocate to ER-PM junctions where it can facilitate SOCE.

SOCE participates in many biological events, including gene expression, cell metabolism, tumor progression. Umemura et al. (2014) found SOCE to contribute to melanoma progression via the CaMKII/Raf-1/ERK signaling pathway. Additionally, STIM2-gated ORAI1 Ca^2+^ channels may regulate melanoma, because Stanisz et al. (2014) demonstrated that ORAI1 and STIM2 are highly expressed and control store-operated Ca^2+^ entry in human melanoma, with silencing of ORAI1 and/or STIM2 inhibiting the ability of proliferation, invasion, and migration of melanoma cells. - These findings indicate that ORAI1 and STIM1/STIM2 are potentially useful therapeutic targets for preventing tumor metastasis. STIM1, rather than ORAI channels also mediates Ca^2+^-cAMP crosstalk and pigmentation via oligomerization (Motiani et al., 2018).

The ORAI1 protein was shown to be mainly confined to the basal epidermal layer where it plays a critical role in controlling epithelial cell proliferation and polarized motility (Vandenberghe et al., 2013). Numaga-Tomita and Putney, 2013 also found the knockdown of either STIM1 or ORAI1 to strongly suppress SOCE, causing impaired expression of keratin1, an early keratinocytes differentiation marker, and the inhibition of normal growth of HaCaT cells in low Ca^2+^. Additionally, decreased SOCE in ORAI1-, ORAI2-, and ORAI1/2-deficient immune cells, such as neutrophils, impairs multiple cellular functions, including phagocytosis, degranulation, leukotriene expression, and reactive oxygen species (ROS) production (Grimes et al., 2020). Similarly, ORAI1 and ORAI2 can form a heteromorphic channel complex, in which ORAI2 attenuates the function of ORAI1 and limits SOCE, while ORAI2 fine-tunes the magnitude of SOCE to modulate immune responses (Vaeth et al., 2017). These observations suggest that ORAI proteins may be associated with the skin barrier and immune functions.

ORAI is also reported to interfere with TRP channels. ORAI1 and TRPV1 were found to associate and move in close proximity to each other at the plasma membrane, and Ca^2+^ entering the cell through TRPV1 channels induced strong calcium-dependent ORAI1 inactivation, which influenced cell migration and wound healing (Bastian-Eugenio et al., 2019). Moreover, Woo et al. (2021) synthesized a chemical derivative of valencene, nootkatol, which could inhibit secretion of collagen-degrading enzymes and matrix metalloproteinase-1 (MMP-1) in keratinocytes via TRPV1 and hyperpigmentation in melanocytes via ORAI1, and as well prevents ultraviolet radiation-induced photoaging. TRPV3/4 and ORAI1 work together in keratinocytes could mediated skin barrier formation, (Saul et al., 2014). This finding also provides a promising strategy in targeting ion channels for preventing photoaging.

### Inositol 1,4,5-Trisphosphate Receptor and Ryanodine Receptor

Ca^2+^ release from intracellular stores, mainly ER/SR, is mediated by intracellular ligand-gated Ca^2+^ release channels. Two closely related families of intracellular Ca^2+^ release channels have been identified: the inositol 1,4,5-trisphosphate receptor (IP_3_R) and the ryanodine receptor (RyR). IP_3_R channels are located in all cell types with the highest densities in the Purkinje cells of cerebellum while the RyR represents primary Ca^2+^ release channel in striated muscle. Due to structural homology and similar physiology, there are many functional similarities between IP_3_R and RyR channels (Serysheva., 2014). In resting cells, cytoplasmic Ca^2+^ concentration is maintained at ∼ 100 nM, which is lower than extracellular Ca^2+^ concentration and Ca^2+^ concentration in ER Ca^2+^ store. The activation of IP_3_R and RyR could increase transitorily cytoplasmic Ca^2+^ concentration.

The activity of IP_3_R channels is regulated by coupled interplay between the binding of its primary ligands, IP_3_ and Ca^2+^. IP_3_ is also a second messenger produced through phosphoinositide turnover in response to many extracellular stimuli such as hormones, growth factors, neurotransmitters, neurotrophins, odorants, and light (Fedorenko et al., 2014). Tu et al. (2005) indicated TRPC1 interacts with PLCgamma1 and IP_3_R, causing the activation of SOC in human keratinocytes. Schmitt et al. (2021) recently demonstrated that the inhibition of phospholipase C (PLC), blocks IP_3_R Ca^2+^ release, ameliorating alterations of autoantibodies targeting Dsg1 and Dsg3 localization and improving blistering of human epidermis in pemphigus. IP_3_ binds to IP_3_R causing the release of Ca^2+^ from intracellular stores and elevation of cytoplasmic free Ca^2+^ concentration, which triggers diverse cellular actions, ranging from contraction to secretion, from proliferation to cell death (Baker et al., 2021). Furthermore, IP_3_R interacts with Ca^2+^ in a biphasic manner, i.e. activation at low concentrations (up to 0.3 μM) and inhibition at higher concentrations (0.5–1 μM) (Bezprozvanny et al., 1991) and IP_3_R activity is also regulated by Ca^2+^-independent accessory proteins, Mg^2+^, redox potential and ATP (Thrower et al., 2001).

**FIGURE 3:**
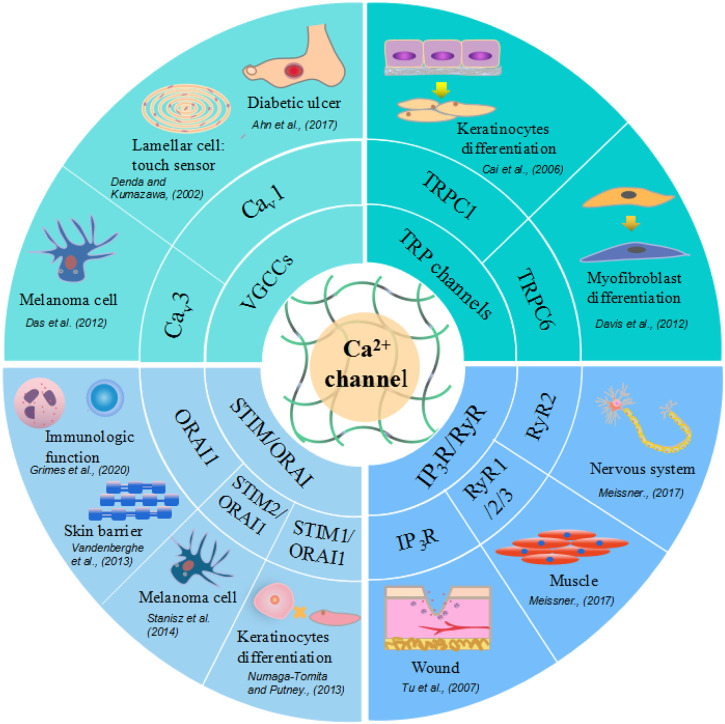
Different calcium ion channels and related skin cells and physiological function. Voltage-Gated Calcium (Ca_v_) Channels are lamellar cell touch sensors of skin, with increased expression in melanoma cells and decreased in diabetic ulcer. TRP channels facilitate keratinocytes and myofibroblast differentiation. STIM or ORAI promote keratinocytes proliferation, skin barrier formation and regulate immunologic function. Ryanodine receptors express in nervous system, muscle, brain tissue and also function in wound healing.

RyRs play an important role in the regulation of intracellular calcium levels in the nervous system and muscle. Sumiko et al. (Denda et al., 2012) reported that RyR1 is strongly expressed in differentiated layers of the epidermis; RyR2 is also expressed in the differentiated layers, especially in the border layer between the stratum corneum and the granular layer; and RyR3 is expressed throughout the epidermis, although its expression is stronger in differentiated layers than in basal layers. A different study showed that the inhibition of RyRs can accelerate wound closure *in vivo*, while in HaCaT cells (keratinocytes), wound closure is accelerated by treatment with dantrolene, a RyR antagonist (Degovics et al., 2019). These results suggest that in the epidermis, RyRs are associated with both the differentiation of epithelial cells and epidermal barrier homeostasis. Moreover, the application of RyR antagonists may have therapeutic potential for wound treatment. RyRs and other Ca^2+^ channels, their effectors, and impacts on wound healing are summarized in Figure3.

## Dermatological Diseases

Given calcium channels and their connection with the skin, it is highly likely that calcium channels, especially TRP channels, are involved in certain skin disorders. There is mounting evidence that aberrant TRP channel expression and function contributes to several skin diseases associated with altered cell differentiation and proliferation (Table 1).

**TABLE 1:** Role of TRP and SOC channels in several dermatologic diseases.

Disease	Volvement of calcium channels	Type	Up/down	Findings and potential mechanisms	Therapeutic approaches	References
Atopic dermatitis	TRPC6	Nonselective	—	Inhibited Ca^2+^ influx, perturbed keratinocytes differentiation	TRPC6 activator, hyperforin might be beneficial	Schempp et al. (2003)
	TRPV1	Weekly Ca^2+^-selective	Up	Increased the release of cytokines and neuropeptides	TRPV1 antagonist PAC-14028 can be a novel antipruritic therapy	Lee et al. (2019)
	TRPV3	Weekly Ca^2+^-selective	Up	Attenuated inflammation factors production and scratching behavior	TRPV3 maybe potential therapeutic target	Seo et al. (2020)
	TRPM8	Nonselective	—	TAC activated TRPM8 currents and gating in lipid bilayers	Tacrolimus (FK506) could treat AD, TRPM8 may be effective	Arcas et al. (2019)
	TRPA1	Nonselective	UP			Nattkemper et al. (2018)
	TRPV2	Nonselective	UP			Nattkemper et al. (2018)
Psoriasis	STIM1	Ca2+ release-activated	Up	Induced chemotactic factors and neutrophil chemotaxis	STIM1 in neutrophils may a target for the amelioration of psoriasis	Steinckwich et al. (2015)
	TRPC1/4/5/6/7	Nonselective	Down	Functioning of SOCCs and capacitative calcium entry is clearly disturbed	Activation or up-regulation might be beneficial	Karvonen et al. (2000)
	TRPV1	Weekly Ca^2+^-selective	Up			Lee et al., 2020
	TRPV3	Weekly Ca^2+^-selective	Up	TRPV1 currents in dissociated DRG neurons via the ALX/FPR2 induced acute pain and itch	Potent inhibitors of TRPV1 may offer new therapies for psoriasi	Nattkemper et al. (2018)
	TRPM8	Nonselective	Up			Nattkemper et al. (2018)
Darier’s disease	TRPC1/4	Nonselective	Up	Partly compensated for Ca^2+^ store depletion in the ER induced by ATP2A2 dysfunction	Increased expression can be compensatory mechanism	Hovnanian, (2004)
Non-melanoma cancers	TRPC1/4	Nonselective	Down	Diminished calcium entry and failed differentiation of BCC cells	Up-regulation may effective	Beck et al. (2008)
	TRPC6	Nonselective	Up	Suppressed cell growth and evoked differentiation	Triterpenes induced TRPC6 expression might a therapy	Woelfle et al. (2010)
	TRPA1	Nonselective	Down			Fusi et al. (2014)
	TRPV4	Ca^2+^-selective		Increased IL-8 production, which in turn downregulated TRPV4 expression	TRPV4 reduction could be early biomarker of skin carcinogenesis	Fusi et al. (2014)
Olmsted syndrome	TRPV3	Weekly Ca^2+^-selective	Up	Missense mutations in TRPV3 elevated apoptosis of keratinocytes and skin hyperkeratosis	Selectively targeting TRPV3 maybe a therapeutic potential	Lin et al. (2012)
Prurigo nodularis	TRPV1	Weekly Ca^2+^-selective	Up	Enhanced substance P, calcitonin gene-related peptide	Activation might be beneficial	Stander et al. (2004)
Skin ageing and UV-induced diseases	TRPV1	Weekly Ca^2+^-selective	Up	Activated PKC and Ca^2+^ influx, regulated cytokines expression	Inhibitor TIP could attenuate injury	Kang et al. (2017)
	TRPC7	Nonselective	Up	Mediated UVB-induced Ca^2+^ influx, ROS production	Reduction in TRPC7 activity Suppressed UVB-induced aging	Hsu et al. (2020)
Burn injury	TRPV1	Weekly Ca^2+^-selective	Up	Promoted inflammatory cell invasion and myofibroblast generation	Absence might be effective	Okada et al. (2011)
	TRPA1	Nonselective	Up	Enhanced inflammatory cytokines and α-SMA expression		Okada et al. (2011)
Diabetic ulcer	L-type VGCC	Ca^2+^-selective	Down	Stimulated nitric oxide (NO) production	Inhibition facilitates diabetic wound healing	Bagheri et al. (2011)

### Atopic Dermatitis

AD is a common chronic inflammatory skin disorder that results from complex interactions between genetic and environmental factors (Liang et al., 2016). The symptoms of this disease include intense itching, frequent exacerbation (Furue and Kadono, 2015), dry skin in characteristic locations, and pruritus (Bos et al., 2010; Wolf and Wolf, 2012). AD is considered to be a biphasic, T cell-mediated disease (Furue and Kadono, 2017). The T helper (Th) 2 signal predominates in the acute phase, whereas a Th2-Th1 switch promotes disease chronicity (Gittler et al., 2012; Guttman-Yassky et al., 2018).

TRPC6 dysfunction in epithelial cells is found to trigger AD pathogenesis through inhibiting Ca^2+^ influx, perturbing cell differentiation, and impairing epidermal barrier functions (Sun et al., 2012). Hyperforin, a TRPC6 activator, could partially restore the impaired differentiation of psoriatic keratinocytes (Leuner et al., 2011). Interestingly, the topical application of hyperforin-containing cream can improve the symptoms of AD (Schempp et al., 2003) (Table 2). TRPC6 has potential as a target for AD treatment; however, further preclinical and clinical studies are required to confirm this. Similarly, TRPM8 was reported to be a pharmacological target for tacrolimus (FK506), a macrolide immunosuppressant, for the treatment of AD (Arcas et al., 2019). Lee et al. (2019) reported a novel, topical, nonsteroidal antagonist of TRPV1, PAC-14028 cream, which could moderate atopic dermatitis with promising efficacy and safety. Moreover, treatment with TRPV1 antagonist also ameliorates barrier integrity, as measured by the levels of *trans*-epidermal water loss and differentiation markers, filaggrin and loricrin (Yun et al., 2011; Yun et al., 2011). Mutations have been identified in the TRPV3 gene, thereby linking TRPV3 to pruritus and AD (Asakawa et al., 2006; Yoshioka et al., 2009). TRPV3 is upregulated in the skin of MC903-induced AD mouse model and the pharmacologic inhibition of TRPV3, attenuates production of inflammation factors and scratching behavior induced by AD (Seo et al., 2020), suggesting that TRPV3 may be a potential therapeutic target for AD. TRPV3 knockout mice exhibit curly whiskers and body fur abnormalities (Cheng et al., 2010), while hairless DS-Nh mice also exhibit signs of spontaneous dermatitis, resembling human AD (Asakawa et al., 2006). Nattkemper et al. (2018) also reported increased expression of genes for TRPV2 and TRPA1 in pruritic atopic skin, and elevated expression of genes for TRPM8 and TRPV3 in pruritic psoriatic skin (Nattkemper et al., 2018). Impaired keratinocyte differentiation is a key feature of AD, whereas inflammatory reaction and TRPC6 activation is sufficient for keratinocyte differentiation. TRPV1, TRPV3, or TRPM8 are responsible for secretion of proinflammation factors and aggravate clinical symptom. This suggests TRPC6, TRPV1, TRPV3 or TRPM8 play a role in development of AD, they may play the parts through different mechanisms and the possibility of existing interaction between them remains unclear. This may account for the different effect of TRPC6 and TRPV1, TRPM8 or TRPV3 on AD.

**TABLE 2:** Summary of related calcium channels drugs.

Target Ca^2+^ channels	Agonist/Antagonist	Drugs	Related skin diseases	Potential pharmacologic effect	References
VGCC	Antagonist	Mibefradil	Melanoma	Induced apoptosis, impairs migration	Barcelo et al. (2020)
	Antagonist	Kurtoxin	Melanoma	Reduce viability and proliferation	Das et al. (2012)
	Antagonist	Nifedipine	Skin barrier	Accelerated the barrier recovery	Denda et al. (2006)
	Antagonist	Verapamil	Skin barrier	Accelerated the barrier recovery	Denda et al. (2006)
	Antagonist	R-(+)-BAY K8644	Skin barrier	Accelerated the barrier recovery	Denda et al. (2006)
TRPV1	Antagonist	TIP	Skin ageing	Prevented UV-induced MMP-1 and pro-inflammatory cytokines	Kang et al. (2017)
	Antagonist	PAC-14028 cream	Atopic dermatitis		Lee et al. (2019)
	Antagonist	AG1529	Neurogenic inflammation	Abolished inflammation, reduced neuronal firing	Nikolaeva-Koleva et al. (2021)
	Antagonist	Capsazepine	Itch		Moran, (2018)
	Antagonist	SB-705498	Itch	Influenced neurogenic inflammation	Holland et al. (2014)
ORAI1	Antagonist	Spirodela polyrhiza	Atopic	Inhibited mast cell degranulation	Nam et al. (2017)
TRPV3	Agonist	Extract	Dermatitis	Partially restore the impaired differentiation of keratinocytes	Nam et al. (2017)
TRPC6	Agonist	Hyperforin-containing cream	Atopic Dermatitis	Ameliorated barrier integrity	Schempp et al. (2003)
TRPA1	Antagonist	HC-030031	Atopic Dermatitis		Oh et al. (2013)
TRPM8	Agonist	Thymol	Psoriasis	Attenuated the enhanced infiltration of dermal immune cells, downregulated expression of pro-inflammatory cytokines	Wang et al. (2020)

### Psoriasis

Psoriasis, a debilitating skin condition, is considered to be an autoimmunity-mediated disease involving several components of the immune system, including neutrophils (Toichi et al., 2000). In a mouse model of psoriasis, targeted knockout of STIM1 in myeloid lineage cells (including neutrophils) hastened the reversal of psoriatic plaques following the removal of a chemical activator of psoriasis (Steinckwich et al., 2015).

TRPM8 activated by thymol could attenuate the enhanced infiltration of dermal immune cells and downregulate expression of pro-inflammatory cytokines (Wang et al., 2020). Another study reported that TRPCs, including TRPC1, TRPC4, TRPC5, TRPC6, and TRPC7, are inhibited in the epidermis *in situ* and in vitro-cultured epithelial cells derived from psoriasis patients (Karvonen et al., 2000). Compared with healthy cells, psoriatic epithelial cells show a diminished response after thapsigargin-mediated calcium store depletion, suggestive of impaired SOCE. Furthermore, exposing cultured psoriatic epithelial cells to high levels of extracellular Ca^2+^ leads to only a minor Ca^2+^ influx, which is most likely due to the impaired function of TRPCs on the cell surface membrane of epithelial cells (Leuner et al., 2011).

### Darier’s Disease

Darier’s disease (DD) (Darier White’s disease, keratosis follicularis), a genetic skin disorder first described in 1889 by Darier and White, is characterized by the loss of intracellular adhesion and disordered keratinization leading to warty plaques and papules in the seborrheic areas (Cooper and Burge, 2003). The causal mutation is located in the ATP2A2 gene that codes for the type 2 SERCA, a protein that regulates intracellular calcium homeostasis by pumping cytosolic calcium back into the ER (Sakuntabhai et al., 1999; Celli et al., 2012). The expression of the SOCCs TRPC1 and TRPC4 increases in response to raised extracellular Ca^2+^ concentrations (Hovnanian et al., 2004). Importantly, epidermal keratinocytes from Darier’s patients and HaCaT keratinocytes in which SERCA2 expression is knocked down through siRNA treatment, display increased TRPC1 expression (Elsholz et al., 2014), which is speculated to be a compensatory mechanism (Pani and Singh, 2008).

### Nonmelanoma Cancers

Skin cancers, the most frequently diagnosed malignancies in humans (Simoes et al., 2015), are broadly divided into melanoma and nonmelanoma types. Basal cell cancers (BCCs) account for 65–70% of all nonmelanoma skin cancers (Wieckiewicz et al., 2013). Additionally, the premalignant form of squamous cell carcinoma (SCC; actinic keratosis), another nonmelanoma skin cancer, accounts for more than 250,000 new cases in the United States annually (Ratushny et al., 2012). Both cancer subtypes originate in the basal layer of the epidermis (Fusi et al., 2014). In BCC, the lack of TRPC1 and TRPC4 protein *in vitro* can lead to diminished calcium entry after calcium-induced differentiation and subsequently to the failed differentiation of BCC cells (Beck et al., 2008). The induction of TRPC6-mediated Ca^2+^ influx by triterpenes in epithelial cells isolated from patients with actinic keratosis (*in situ* SCC) suppresses cell growth and promotes differentiation (Woelfle et al., 2010). Fusi et al., 2014 showed that TRPA1 protein and mRNA expression levels are significantly increased in skin biopsies from patients with solar keratosis, a premalignant form of nonmelanoma skin cancer. Other studies have shown that TRPV4 stimulation leads to the release of IL-8, which in turn, downregulates TRPV4 expression in a human keratinocyte cell line (HaCaT), and the selective reduction of TRPV4 expression may represent an early biomarker of skin carcinogenesis (Fusi et al., 2014).

### Olmsted Syndrome

Olmsted syndrome is a rare genodermatosis belonging to the heterogeneous group of palmoplantar keratodermas (PPKs) (Duchatelet and Hovnanian, 2015). The clinical symptoms are variable, but typically severe and disabling (Kariminejad et al., 2014). Lin et al. identified that genetic mutations that alter the same (Gly573Ser, Gly573Cys) or a different residue (Trp692Gly) in TRPV3 are responsible for Olmsted syndrome in humans. Increased intracellular calcium concentrations can cause apoptosis and consequently, the characteristic hyperkeratosis seen in Olmsted syndrome patients (Lin et al., 2012). However, it has been speculated that the pathophysiology of Olmsted syndrome might not be explained solely by abnormal TRPV3 function in keratinocytes, but may also involve immune dysfunction arising in other cells such as cutaneous Langerhans cells (Danso-Abeam et al., 2013).

### Prurigo Nodularis

Prurigo nodularis (PN) is a chronic disorder of the skin that is commonly characterized by the presence of multiple, firm, flesh-to-pink colored nodules on the extensor surfaces of the extremities (Stander et al., 2020). Continuous scratching is a major symptom, while itching and scratching of the lesions contribute to the cycle that makes this disease difficult to treat, thus reducing the quality of life of affected patients (Tsianakas et al., 2016). Although TRPV1 was identified as being highly expressed in pathological skin lesions of PN patients (Stander et al., 2004), as well as in UV-irradiated photo-aged and intrinsically aged skin (Lee et al., 2009). The exact mechanism underlying the role of TRPV1 in PN remains unclear.

### Skin Aging and UV-Induced Diseases

Although the skin is incredibly durable and has an enormous regenerative capacity, it cannot escape aging due to the turnover rate of epidermal cells (Rinnerthaler et al., 2015). Skin aging leads to, among other effects, deleterious alterations in the structure and function of dermal collagen (Quan et al., 2021). With aging, the structure and function of the skin barrier changes. Some of the changes include, significant increase in the number of SC layers, impairment of SC integrity, delayed recovery after acute perturbation with tape stripping and the epidermis becomes thinner (Choi, 2019). The epidermal calcium gradient which acts as a regulatory signal for skin barrier homeostasis, also become disrupted. Many factors, both internal and external, are responsible for skin aging-associated changes.

Glycolic acid (GA) pretreatment, along with UVB irradiation, can synergistically induce TRPV1 expression in human keratinocytes (Tang et al., 2019), leading to Ca^2+^ entry and consequently, an enhanced release of cytoplasmic calcium and ER stress (Lai et al., 2011). The regulation of the performance of TRPV1 is still not clear. Lee, Y.M., et al. (2009) reported that TRPV1 expression in human skin epithelial cells can be increased *in vivo* by UV, concomitant with an upregulation of MMP-1 expression; the latter might be mediated at least in part, by PKC-dependent activation of TRPV1 and subsequent Ca^2+^ influx (Lee et al., 2009). Moreover, a novel TRPV1-inhibiting peptide was demonstrated to attenuate UV-induced erythema and the expression of MMP-1, MMP-2, IL-6, and IL-8 in human skin *in vivo* (Kang et al., 2017). Recently, a study found TRPC7 mediated UVB-induced Ca^2+^ influx, UVB-induced ROS production, and UVB-induced epidermal aging in mice (Hsu et al., 2020). These findings suggest that inhibition of specific TRP channels may delay skin aging. Whether other calcium ion channels are related to aging is yet to be discovered in further studies.

### Burn Injury

Burn injuries are associated with substantial morbidity and mortality. Although burn injuries can be caused by friction, cold, heat, radiation, and chemical or electric sources, most are caused by heat from hot liquids, solids, or fire. Generally, burn injuries, particularly severe burns, trigger numerous physiological and pathophysiological responses such as metabolic changes, distributive shock, and inflammatory responses (Jeschke et al., 2020). Following burn injury, calcium will be released from ER calcium stores mediated by increased IP_3_R activity (Jeschke et al., 2009). TRPV1 is a noxious heat sensor and is associated with mechanical and thermal hyperalgesia after burn injury. The overexpression of fibulin-5, which can promote dermal wound healing, is shown to attenuate burn injury-induced inflammatory responses via the suppression of the TRPV1/CGRP pathway (Hou et al., 2017). Similarly, the expression of TRPV1 and TRPA1 is reported to be upregulated in epithelial cells of an alkali-burned cornea, and the absence of the TRPV1 and TRPA1 genes suppresses post-alkali burn inflammation and facilitates the final healing (Okada et al., 2011; Okada et al., 2014). These results suggest that TRPV1 and TRPA1 play a negative role in burn wound healing, although TRPV1/TRPM3/TRPA1 triple knockout mice lack the acute withdrawal response to noxious heat that is necessary to avoid burn injury (Vandewauw et al., 2018).

### Diabetic Ulcer

Diabetes mellitus (DM) is fast becoming a lifestyle-related pandemic. Leg or foot ulcers are the most commonly occurring wounds in diabetic patients (Patel et al., 2019), and can lead to amputation in the most extreme cases. In a model of type-1 DM, the expression of Ca_v_1.2 acting as a cellular calcium channel and plasma membrane Ca^2+^-ATPase (PMCA), one of the calcium transport-related factors decreases, indicating that the concentration of intracellular calcium gradually depletes (Ahn et al., 2017). Another study showed that azelnidipine (AZL), a new calcium channel blocker with selectivity for L-type voltage-operated calcium channels, facilitates diabetic wound healing via stimulating nitric oxide (NO) production and enhancing processes central to normal wound healing (Bagheri et al., 2011). Although several studies have highlighted the role of TRP channels in pancreatic β cells and in the regulation of insulin secretion (Philippaert and Vennekens, 2017), the potential influence of TRP channels in diabetic ulcers remains unclear and merits further investigation.

## Concluding Remarks

As a signal of survival and death, Ca^2+^ regulate almost all physiological activities and impact nearly every aspect of cellular life. Calcium channels are participants and regulators in the maintenance of calcium dynamic balance and epidermal barrier. The roles of calcium channels in skin homeostasis and different dermatologic diseases have been gradually realized. Different types of calcium channel mediate Ca^2+^ influx and have similar or distinct function. Various calcium channels are connected with dermatologic diseases and several targeting drugs. The up- or down-regulation of Ca^2+^ channels may favor recovery of several dermatologic diseases. However, the exact mechanisms mediating the complex roles of relevant Ca^2+^ channels in dermatologic diseases have not yet been fully elucidated. Ca^2+^ channels would be effective molecules for targeted therapy and ideal biomarkers for skin cancer diagnosis. Although the role and potential of Ca^2+^ channels have been investigated in various skin injury and diseases, there are still a number of unknowns surrounding their interactions and skin repair mechanisms, providing focus for further future studies.

Here we highlight only few of them:

•It is not quite clear how the calcium current is changed during the wound healing process?
•How are TRP channels initially opened following wounding and whether the altered expression or function is a cause or a consequence?
•Does Ca^2+^ channels associate with triggering inflammation and/or switching off the wound healing response?

Ca^2+^ channels function both in excitable cells, such as neurons, smooth muscle cells via sensing potentials change potentially and the non-excitable cells, like skin cells. How do Ca^2+^ channels regulate differently on the distinct two types of cells?

There also exists a class of drugs, called calcium channel blockers (or calcium antagonists) that decrease the flow of Ca^2+^ through calcium channels and agonists which have the opposite effect. Whether it could be a therapy strategy?

In addition to the Ca^2+^channels mediating the influx of extracellular Ca^2+^into the cytoplasm, some transporters such as Ca^2+^pumps, cell membrane sodium-calcium exchangers, and endoplasmic reticulum Ca^2+^pumps, regulate the release of Ca^2+^from cell or influx into organelles. Calcium sensing receptors are critical to maintenance of organismal Ca^2+^ homeostasis. However, they sense not only Ca^2+^, but also the metabolic environment. They can be regulated by a variety of metabolic signals, including amino acids, polyamines, pH, cAMP, polyvalent cations, ionic strength, making CaSR capable of generating cell- and tissue-specific responses (Breitwieser et al., 2004). There exist other specific ion channels and transporters involved in the regulation of cellular calcium signaling in different cells. Various calcium channels, calcium transporters, and calcium receptors, interact with each other to maintain calcium homeostasis, and the dysfunction of any link may lead to the occurrence of disease. Due to different location, distribution, permeability, selectivity, dynamics and regulation factors of ion channels, the calcium signals generated have different temporal and spatial characteristics and different physiological functions.

Future studies of Ca^2+^ channels will not only shed lights on their roles in dermatologic diseases but will open new avenues for possible mechanism and further candidate drugs for therapeutics.
